# Understanding health seeking behaviors to inform COVID-19 surveillance and detection in resource-scarce settings

**DOI:** 10.7189/jogh.10.0203106

**Published:** 2020-12

**Authors:** Bach Xuan Tran, Giang Thu Vu, Huong Thi Le, Hai Quang Pham, Hai Thanh Phan, Carl A Latkin, Roger CM Ho

**Affiliations:** 1 Institute for Preventive Medicine and Public Health, Hanoi Medical University, Hanoi, Vietnam; 2 Bloomberg School of Public Health, Johns Hopkins University, Baltimore, Maryland, USA; 3 Center of Excellence in Evidence-based Medicine, Nguyen Tat Thanh University, Ho Chi Minh City, Vietnam; 4 Institute for Global Health Innovations, Duy Tan University, Da Nang, Vietnam; 5 Faculty of Pharmacy, Duy Tan University, Danang, Vietnam; 6 Faculty of Medicine, Duy Tan University, Da Nang, Vietnam; 7 Institute for Health Innovation and Technology (iHealthtech), National University of Singapore, Singapore, Singapore; 8 Department of Psychological Medicine, National University Hospital, Singapore, Singapore

As the COVID-19 pandemic continues its deadly reign all over the world, devising and implementing effective strategy for detecting and controlling the infection has become ever more critical. While a number of developed countries have utilized mass community testing of suspected infection to effectively manage the spread and severity of the pandemic, less-developed nations struggle to implement similar measure due to various financial and human resource constrains faced by their health system. In this viewpoint, we discuss how by understanding health seeking behavior of a country’s population, developing countries can identify and set priorities to most resource-efficient disease management measures, which then would help them achieve successes in controlling COVID-19 in their countries. The viewpoint concludes with an example of such success cases.

As of July 18, 2020, there have been over 13.8 million confirmed cases of SARS-COV-2 with death toll amounted to 593 087 [1]. A number of countries, mostly developed nations with long-considered advanced health care system have started rolling out mass community testing for the virus, alongside with restrictions on population mobility. Some experts have argued that population-wide testing is a more reliable and reasonable way of detecting and controlling the infection – as the economy and individual’s mental health suffer the side effects of quarantine and social distancing, while the uncertainty of when the disease will peak and what follows is still looming [2]. The situation is more complicated, though, for developing nations with frequently over-burdened health system and rather diverse health seeking behaviors, such that community-wide testing per se may not be the most appropriate and effective choice.

The lack of health professionals, limited financial resources for health care, and the under-developed health infrastructure may all be challenges in implementing mass testing of SARS-COV-2 in the community in developing countries. In a time of need for pandemic response, even mobilization of resources and support from donations may not help overcome these problems completely. Setting priorities to target interventions by identifying suspected cases in various geographical locations requires the understanding of health seeking behaviors among local communities. While most people in Western countries visit health clinics or family doctors when perceiving a health problem, previous studies have described various contextual factors and barriers that shape access and utilization of health care services in resource-scare settings [3,4]. Many residents prefer to purchase un-prescribed medications at pharmacies for self-treatment, or visit traditional healers, private or non-registered clinics, rather than hospitals and official health stations as places of first contact for health issues (for example, people in Pakistan regions (39.1%) and Indonesia regions (42.9%) preferred going to pharmacy first to treat illness; **Table 1**). This would be a larger obstacle to confirming and monitoring cases, especially in the early stages of COVID-19 epidemics, including cases importation and cluster transmission.

**Table 1:** Examples of health seeking behaviors in countries with frequent local epidemics (Unit: %)

Ref.	Countries	District	Health problem/population	Medical health providers						Pharmacy	Self-treatment	Others	Not seeking
**Grass-root**	**Commune health station**	**Hospital central**	**Private hospital**	**Private clinics**	**Traditional medicine worker**
[5]	Indonesia	Papua	Malaria	32.2			37.8		·	6.1		.	24.0
[6]	Indonesia	West Java	Fatal illnesses in young children	36.0					42.4				21.6
[7]	Indonesia	Jogjakarta	Tuberculosis	40.8					40.8				11.3
[8]	Uganda	Kampala	Chronic cough	59.6			25.0		0.6	13.5		1.3	
[9]	Pakistan	Islamabad	Students	26.6			73.4						
[10]	Pakistan	Rawalpindi, Islamabad, Abbotabad, Peshawar	General problem	18.4			26.2			39.1		23.5	
[11]	Indonesia	West Java	Rural population	16.6			12.6		5.7	42.9	20.5		
[12]	Pakistan	Karachi	Terminal child illness	14.0			15.0	68.0	3.0				
[13]	Bangladesh	Bangladesh	Childhood acute respiratory tract infections	12.5			24.7		26.3	26.4			10.3
[14]	Ethiopia	Gambella	Sexually transmitted infections	Rank 2*						Rank 1			56.8
[15]	Indonesia	South Sulawesi	Elderly health problem	Rank 1		Rank 3	Rank 2						
[16]	South Africa	Johannesburg	Common infectious	Rank 2		Rank 3		Rank 2	Rank 4	Rank 1		Rank 5	
[17]	China	Hong Kong	Respiratory and gastrointestinal-related infections	Rank 3			Rank 2		Rank 1				
[18]	Guatemala	Chimaltenango, Totonicapán, Suchitepequez Jalapa	Child illness	Rank 2			Rank 3		Rank 4	Rank 1			

Ref. – reference

*For publication where no indication of percentage (%) of participant using a provider is found, we ranked the providers in terms of time of contact (ie, first contact will be Rank 1)

People with mild COVID-19 symptoms that in many cases are similar to a common or seasonal cold, do not thinking that they may have been infected with the virus, and may go to these non-official health facilities for medication, increasing the risk of exposure of others while limiting the chance of tracing back to first infection case (F0). They may be long gone before other positive cases infected by them are detected. In addition, people who believed they might have been infected based on their symptoms may also ask their families and friends to get their medication from health workers in the communities or from the pharmacies, rather than going to hospitals or testing centers. Such behavior is likely to be induced by the fear of stigma towards them, should they be tested positive, as well as fear of having their whole families transferred to quarantine location, or having to disclose their past activities for contact tracing. SARS-COV-2 associated stigma, which can undermine the testing and monitoring efforts, has been one of the major concerns of health experts and organizations globally [19]. The habit and ease of seeking health advice and medication from pharmacies, traditional health providers, and private/non-official clinics in developing countries is likely to exacerbate such problem.

**Figure:**
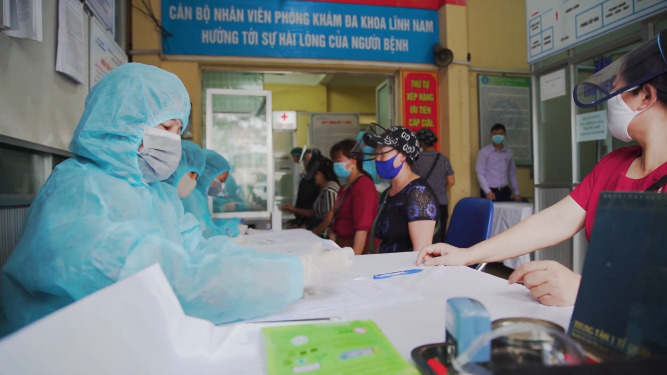
Photo: Rapid COVID-19 testing registration desk in Hanoi (from: Truyền Hình Pháp Luật, via https://commons.wikimedia.org/wiki/File:Vietnamese_registered_for_rapid_testing_(COVID-19).png).

To effectively detect and control the SAR-COV-2 infection in these resource-scarce settings, thus, would require the active and thorough involvement of health facilities other than hospitals and official health centers, especially in more remote regions where accessibility to official health care is limited. Pharmacies, traditional healers, village health collaborators, private clinics, or mobile independent health workers in the commune should be considered as gatekeepers in a closely connected network of COVID-19 surveillance. Ideally, a well-determined mechanism for timely information sharing between these first contact points and higher-level and specialized taskforces should be established. Staff at these facilities should be trained to detect signs and epidemiological history of suspected COVID-19 cases from or relating to their customers while being provided with sufficient equipment for their own disease protection. These local health gatekeepers can also be effective, community-based, and far-reaching channels in which accurate information regarding COVID-19 knowledge and response can be delivered to the individuals. For example, pharmacists can persuade disease-suspecting customers to visit hospital or testing centers. In addition, due to their proximity to the residency and familiarity with local residents, these facilities would also be points via which intervention packages being delivered to the community, in the unfortunate case of prolonged disease.

One of the examples of how understanding health seeking behavior of population can result in effective strategies for detecting and controlling SARS-COV-2 infections is the case of Vietnam. A low middle income country with health system facing numerous constrains, Vietnam has so far managed to keep the number of SARS-COV-2 confirmed infected cases at 382 and no mortality as of 18 July 2020, through effective utilization of the network of non-official, community-based health facilities and pharmacies, based on the knowledge that majority of the Vietnamese population would prefer going to these local, non-official health workforce when having health problems [20]. We believe that this current success story would further encourage similar resource-scarce settings all over the world to pay more attention to health seeking behaviors of their population and effects of such behaviors on disease management when developing and implementing COVID-19 surveillance and detection measures.

## References

[1] WHO. Coronavirus (Covid-19). 2020. Available: https://who.sprinklr.com/. Accessed: 26 May 2020.

[2] Studdert DM Hall MA Disease Control, Civil Liberties, and Mass Testing — Calibrating Restrictions during the Covid-19 Pandemic. N Engl J Med. 2020;383:102-4. 10.1056/NEJMp2007637 32272003

[3] Ibeneme S Eni G Ezuma A Fortwengel G Roads to Health in Developing Countries: Understanding the Intersection of Culture and Healing. Curr Ther Res Clin Exp. 2017;86:13-8. 10.1016/j.curtheres.2017.03.001 29234482 PMC5717292

[4] Kok MC Kane SS Tulloch O Ormel H Theobald S Dieleman M How does context influence performance of community health workers in low- and middle-income countries? Evidence from the literature. Health Res Policy Syst. 2015;13:13. 10.1186/s12961-015-0001-3 25890229 PMC4358881

[5] Karyana M Devine A Kenangalem E Burdarm L Poespoprodjo JR Vemuri R Treatment-seeking behaviour and associated costs for malaria in Papua, Indonesia. Malar J. 2016;15:536. 10.1186/s12936-016-1588-8 27821127 PMC5100266

[6] Sutrisna B Reingold A Kresno S Harrison G Utomo B Care-seeking for fatal illnesses in young children in Indramayu, west Java, Indonesia. Lancet. 1993;342:787-9. 10.1016/0140-6736(93)91545-W 8103880

[7] Ahmad RA Richardus JH de Vlas SJ Care-seeking behaviour among individuals with TB symptoms in Jogjakarta Province, Indonesia: a community-based study. Int Health. 2013;5:51-7. 10.1093/inthealth/ihs002 24029846

[8] Muttamba W Ssengooba W Kirenga B Sekibira R Walusimbi S Katamba A Health seeking behavior among individuals presenting with chronic cough at referral hospitals in Uganda; Missed opportunity for early tuberculosis diagnosis. PLoS One. 2019;14:e0217900. 10.1371/journal.pone.0217900 31170234 PMC6553765

[9] Manzoor I Hashmi NR Mukhtar F Determinants and pattern of health care services utilisation in post graduate students. J Ayub Med Coll Abbottabad. 2009;21:100-5. 20929025

[10] Hussain S Malik F Hameed A Ahmad S Riaz H Exploring health seeking behavior, medicine use and self medication in urban and rural Pakistan. South Med Rev. 2010;3:32-4.

[11] Berman P Ormond BA Gani A Treatment use and expenditure on curative care in rural Indonesia. Health Policy Plan. 1987;2:289-300. 10.1093/heapol/2.4.289

[12] Hasan IJ Khanum A Health care utilization during terminal child illness in squatter settlements of Karachi. J Pak Med Assoc. 2000;50:405-9. 11191439

[13] Sultana M Sarker AR Sheikh N Akram R Ali N Mahumud RA Prevalence, determinants and health care-seeking behavior of childhood acute respiratory tract infections in Bangladesh. PLoS One. 2019;14:e0210433. 10.1371/journal.pone.0210433 30629689 PMC6328134

[14] Tsadik M Lam L Hadush Z Delayed health care seeking is high among patients presenting with sexually transmitted infections in HIV hotspot areas, Gambella town, Ethiopia. HIV AIDS (Auckl). 2019;11:201-9. 10.2147/HIV.S210977 31564990 PMC6724613

[15] Irwan AM Kato M Kitaoka K Kido T Taniguchi Y Shogenji M Self-care practices and health-seeking behavior among older persons in a developing country: Theories-based research. Int J Nurs Sci. 2016;3:11-23. 10.1016/j.ijnss.2016.02.010

[16] Mapuroma R Cohen C Kuonza L Musekiwa A Tempia S Tshangela A Healthcare seeking behaviour for common infectious syndromes among people in three administrative regions of Johannesburg, South Africa, 2015: a cross-sectional study. Pan Afr Med J. 2019;33:159. 10.11604/pamj.2019.33.159.18461 31565121 PMC6756806

[17] Zhang Q Feng S Wong IOL Ip DKM Cowling BJ Lau EHY A population-based study on healthcare-seeking behaviour of persons with symptoms of respiratory and gastrointestinal-related infections in Hong Kong. BMC Public Health. 2020;20:402. 10.1186/s12889-020-08555-2 32220247 PMC7099823

[18] Goldman N Heuveline P Health-seeking behaviour for child illness in Guatemala. Trop Med Int Health. 2000;5:145-55. 10.1046/j.1365-3156.2000.00527.x 10747275

[19] UNICEF. Social stigma associated with the coronavirus disease (COVID-19). 2020.

[20] WHO-China Joint Mission. Report of the WHO-China Joint Mission on Coronavirus Disease 2019 (COVID-19). Available: https://www.who.int/docs/default-source/coronaviruse/who-china-joint-mission-on-covid-19-final-report.pdf. Accessed: 26 May 2020.

